# Mechanism of Peptide Agonist Binding in CXCR4 Chemokine Receptor

**DOI:** 10.3389/fmolb.2022.821055

**Published:** 2022-03-11

**Authors:** Shristi Pawnikar, Yinglong Miao

**Affiliations:** Center for Computational Biology and Department of Molecular Biosciences, University of Kansas, Lawrence, KS, United States

**Keywords:** chemokine receptors, peptide, agonists, drug design, peptide Gaussian accelerated molecular dynamics, enhanced sampling

## Abstract

Chemokine receptors are key G-protein-coupled receptors (GPCRs) that control cell migration in immune system responses, development of cardiovascular and central nervous systems, and numerous diseases. In particular, the CXCR4 chemokine receptor promotes metastasis, tumor growth and angiogenesis in cancers. CXCR4 is also used as one of the two co-receptors for T-tropic HIV-1 entry into host cells. Therefore, CXCR4 serves as an important therapeutic target for treating cancers and HIV infection. Apart from the CXCL12 endogenous peptide agonist, previous studies suggested that the first 17 amino acids of CXCL12 are sufficient to activate CXCR4. Two 17-residue peptides with positions 1–4 mutated to RSVM and ASLW functioned as super and partial agonists of CXCR4, respectively. However, the mechanism of peptide agonist binding in CXCR4 remains unclear. Here, we have investigated this mechanism through all-atom simulations using a novel Peptide Gaussian accelerated molecular dynamics (Pep-GaMD) method. The Pep-GaMD simulations have allowed us to explore representative binding conformations of each peptide and identify critical low-energy states of CXCR4 activated by the super versus partial peptide agonists. Our simulations have provided important mechanistic insights into peptide agonist binding in CXCR4, which are expected to facilitate rational design of new peptide modulators of CXCR4 and other chemokine receptors.

## Introduction

Chemokine receptors are key G-protein-coupled receptors (GPCRs) which control cell migration during immune system responses, development of cardiovascular and central nervous systems, and in diseases including inflammation and cancer (Balkwill, 2004; Koelink et al., 2012). Particularly, the CCR5 and CXCR4 chemokine receptors function as co-receptors that facilitate HIV entry into host cells (Wu et al., 2010; Qin et al., 2015; Zheng et al., 2017). Antagonists of the CCR5 receptor, named Maraviroc and Vicriviroc, have been used as clinical drugs that could block HIV entry and its replication (Tan et al., 2013). However, ultimately resistance develops due to emergence of viruses that can utilize the CXCR4 co-receptor.

CXCR4 is widely expressed in different human tissues. The primary endogenous chemokine-binding (orthosteric) site is conserved across different subtypes of the chemokine receptors. As a result, development of the CXCR4 antagonists as effective drugs of HIV infection has been greatly hindered due to off-target side effects (Isberg et al., 2016). Consequently, it is appealing to develop allosteric modulators, which selectively bind to a topographically distant (allosteric) site with divergent sequences. They are promising to regulate the responsiveness of CXCR4 to endogenous chemokine with reduced side effects (Christopoulos, 2002; Lolis et al., 2005; Ehrlich et al., 2013).

The endogenous peptide agonist of CXCR4, CXCL12 (SDF-1), drives downstream signaling pathways such as activation of G proteins and mitogen activated protein kinases (MAPK), calcium flux and recruitment of β-arrestin2 (Teicher and Fricker, 2010). The N-terminal amino acids of CXCL12 have been suggested to be critical determinants of the peptide binding and signaling in CXCR4. The first 17 amino acids of CXCL12 are sufficient to activate the receptor, and N-terminal peptides of 9–17 residues can function as a weak agonist of CXCR4 (Heveker et al., 1998; Loetscher et al., 1998). In previous studies, peptides were designed with randomized amino acids at positions 1–4 and residues 5–17 from wild type CXCL12 to identify structural determinants of CXCR4-CXCL12 interactions (Sachpatzidis et al., 2003). Two peptides with residues ASLW and RSVM at positions 1–4 functioned as super and partial allosteric agonists of CXCR4, respectively (Ehrlich et al., 2013). However, molecular mechanism of the allosteric peptide agonist binding to the CXCR4 remains unknown.

Molecular dynamics (MD) serves as a “computational microscope” that can be used to visualize the dynamic behavior of biomolecules over time (Karplus and McCammon, 2002). New algorithms and computing hardware (e.g., Anton supercomputers and GPUs) have been developed over the last several decades, which have enabled less expensive and longer MD simulations (Harvey et al., 2009; Shaw et al., 2010; Johnston and Filizola, 2011; Lane et al., 2013; Hollingsworth and Dror, 2018). Conventional MD (cMD) simulations performed on often the microsecond timescale have proven useful in modeling complex biological processes such as protein-peptide interactions (Ahmad et al., 2008; Dror et al., 2011; Shan et al., 2011; Kruse et al., 2012). However, cMD is still limited for simulations of slower biological processes over longer timescales (e.g., milliseconds and beyond) (Johnston and Filizola, 2011).

Many enhanced sampling methods have been developed to address the above problem (Christen and van Gunsteren, 2008; Abrams and Bussi, 2014; Spiwok et al., 2015; Miao and McCammon, 2016b). In particular, Gaussian accelerated MD (GaMD) is an unconstrained enhanced sampling technique that works by adding a harmonic boost potential to reduce energy barriers. GaMD does not require predefined collective variables or reaction coordinates. Proper reweighting of GaMD simulations can be achieved through cumulant expansion to the second order (Miao et al., 2014). GaMD has been successful to capture complex biological processes including ligand binding (Miao et al., 2015; Miao and McCammon, 2016a; Miao and McCammon, 2017; Pang et al., 2017; Wang and Chan, 2017; Chuang et al., 2018; Liao and Wang, 2018; Miao et al., 2018), protein-protein/membrane/nucleic acid interactions (Palermo et al., 2017; Miao and McCammon, 2018; Park et al., 2018; Sibener et al., 2018; Bhattarai et al., 2019; Ricci et al., 2019; Pawnikar and Miao, 2020), protein folding (Miao et al., 2015; Pang et al., 2017) and GPCR activation (Miao and McCammon, 2016a) through hundreds-of-nanosecond to microsecond timescale simulations. Recently, development of innovative algorithms such as Peptide GaMD (Pep-GaMD) (Wang and Miao, 2020) has greatly expanded our abilities to simulate peptide-protein binding processes. Microsecond Pep-GaMD simulations have successfully captured repetitive binding and dissociation of highly flexible peptides, thereby allowing for highly efficient calculations of peptide binding thermodynamics and kinetics (Wang and Miao, 2020).

High-resolution crystal structures have been determined for the CXCR4 bound by different antagonists such as the small molecule IT1t (Wu et al., 2010), cyclic peptide CVX15 (Wu et al., 2010) and viral chemokine vMIP-II (Qin et al., 2015). The binding pocket of CXCR4 is categorized into a major subpocket, occupied by CVX15, and a minor subpocket, occupied by IT1t and vMIP-II (Qin et al., 2015). Notably, vMIP-II forms a large number of interactions with the receptor N-terminus (Qin et al., 2015). Although these structures provide critical insights into the CXCR4-antagonist interactions, functional mechanisms of chemokine receptors remain poorly understood. Firstly, structures of chemokine receptors in complex with the endogenous chemokines have not been resolved yet. Secondly, the long receptor N-terminus which is critical for endogenous ligand binding affinity and specificity appears disordered in the crystal structures (Suzuki et al., 1994; Brelot et al., 2000; Rajagopalan and Rajarathnam, 2004; Gustavsson et al., 2019). In this context, a complete computational model of CXCR4 bound by the CXCL12 endogenous agonist was recently generated through crosslinking-guided geometry and molecular modeling (Ngo et al., 2020).

In a previous study, we have elucidated binding mode of the Plerixafor (PLX) drug as an antagonist in the CXCR4 receptor through microsecond GaMD simulations. The simulations have also revealed an important intermediate drug-binding site located between the receptor extracellular loop (ECL) 2 and ECL3 for designing novel allosteric modulators. Here, using the recently published computational model of the CXCR4-CXCL12 complex (Ngo et al., 2020), we investigate binding mechanisms of the super and partial peptide agonists in the CXCR4 through novel Pep-GaMD simulations (Wang and Miao, 2020). Representative binding conformations have been determined for each peptide. Low-energy conformational states have also been identified from free energy landscapes of CXCR4 bound by the super and partial peptide agonists. The obtained mechanistic insights provide a significant framework for design of new peptide modulators targeting the CXCR4 receptor.

## Materials and Methods

### Gaussian Accelerated Molecular Dynamics

GaMD is an unconstrained enhanced sampling approach that works by adding a harmonic boost potential to smooth the potential energy surface of biomolecules to reduce energy barriers (Miao et al., 2015). Brief description of the method is provided here.

Consider a system with *N* atoms at positions $\vec{r}=\;\left\{{\vec{r}}_{1}\;,\ldots ,{\vec{r}}_{N}\right\}$ . When potential energy of the system $V\left(\vec{r}\right)$ is less than a threshold energy *E*, a boost potential $\Delta V\left(\vec{r}\right)$ is added to the system as follows:

$$V^{*}\left(\vec{r}\right)=V\left(\vec{r}\right)+\;\Delta V\left(\vec{r}\right),\;V\left(\vec{r}\right)<E$$ (1)

$$\Delta V\left(\vec{r}\right)=\;\frac{1}{2}k{\left(E-V\left(\vec{r}\right)\right)}^{2},\;V\left(\vec{r}\right)<E,$$ (2)

where *k* is the harmonic force constant. The two adjustable parameters *E* and *k* can be determined by application of three enhanced sampling principles. First, for any two arbitrary potential values $V_{1}\left(\vec{r}\right)$ and $V_{2}\left(\vec{r}\right)$ found on the original energy surface, if $V_{1}\left(\vec{r}\right)<$ $V_{2}\left(\vec{r}\right)$ , ΔV should be a monotonic function that does not change the relative order of the biased potential values, i.e., $V_{1}^{*}\left(\vec{r}\right)<$ $V_{2}^{*}\left(\vec{r}\right)$ . Second, if $V_{1}\left(\vec{r}\right)<$ $V_{2}\left(\vec{r}\right)$ , the potential difference observed on the smoothed energy surface should be smaller than that of the original, i.e., $V_{2}^{*}\left(\vec{r}\right)-V_{1}^{*}\left(\vec{r}\right)<\;V_{2}\left(\vec{r}\right)-V_{1}\left(\vec{r}\right)$ . By combining the first two criteria and plugging in the formula of $V^{*}\left(\vec{r}\right)$ and  ΔV , we obtain:

$$V_{max}\leq E\leq V_{min}+\frac{1}{k},$$ (3)

where $V_{min}$ and $V_{max}$ are the system minimum and maximum potential energies. To ensure that Eq. 3 is valid, *k* has to satisfy: $k\leq \frac{1}{V_{max}-V_{min}}$ . Let us define $\;k\equiv \frac{k_{0}}{V_{max}-V_{min}}$ , then $\;0<k_{0}\leq 1$ . Third, the standard deviation (SD) of *∆V* needs to be small enough (i.e., narrow distribution) to ensure accurate reweighting using cumulant expansion to the second order: $\sigma_{\Delta V}=k\left(E-V_{avg}\right)\sigma_{V}\leq \sigma_{0}$ , where $V_{avg}$ and $\sigma_{V}$ are the average and SD of ∆V with $\sigma_{0}$ as a user-specified upper limit (e.g., $10k_{B}T$ ) for accurate reweighting. When *E* is set to the lower bound $E=V_{max}$ according to Eq. 3, $k_{0}$ can be calculated as:

$$k_{0}=\min\left(1.0,k_{0}^{\prime }\right)=min\left(1.0,\frac{\sigma_{0}}{\sigma_{V}}.\frac{V_{max}-V_{min}}{V_{max}-V_{avg}}\right)$$ (4)

Alternatively, when the threshold energy *E* is set to its upper bound $E=V_{min}+\frac{1}{k}$ , $k_{0}$ is set to:

$$k_{0}=k_{0}^{"}\equiv \left(1-\frac{\sigma_{0}}{\sigma_{V}}\right).\frac{V_{max}-V_{min}}{V_{avg}-V_{min}}$$ (5)

if $k_{0}^{"}$ is calculated between 0 and 1. Otherwise, $k_{0}$ is calculated using Eq. 4.

### Peptide Gaussian Accelerated Molecular Dynamics

Peptides often undergo large conformational changes during binding to target proteins, being distinct from small-molecule ligand binding or protein-protein interactions (PPIs). In this regard, Peptide GaMD or “Pep-GaMD” has been developed to enhance sampling of peptide binding (Wang and Miao, 2020). In Pep-GaMD, we consider a system of peptide *L* binding to a protein *P* in a biological environment *E*. Presumably, peptide binding mainly involves in both the bonded and non-bonded interaction energies of the peptide since peptides often undergo large conformational changes during binding to the target proteins. Thus, the essential peptide potential energy is $V_{L}\left(r\right)=V_{LL,b}\left(r_{L}\right)+V_{LL,nb}\left(r_{L}\right)+\;V_{PL,nb}\left(r_{PL}\right)+V_{LE,nb}\left(r_{LE}\right)$ . In Pep-GaMD, we add boost potential selectively to the essential peptide potential energy according to the GaMD algorithm:

$$\Delta V_{L}\left(r\right)=\{\begin{array}{c} \frac{1}{2}k_{L}{\left(E_{L}-V_{L}\left(r\right)\right)}^{2},\;\;V_{L}\left(r\right)<E_{L}\\ 0,\;V_{L}\left(r\right)\geq E_{L} \end{array}$$ (6)

where *E*_*L*_ is the threshold energy for applying boost potential and *k*_*L*_ is the harmonic constant. In addition to selectively boosting the peptide, another boost potential is applied on the protein and solvent to enhance conformational sampling of the protein and facilitate peptide rebinding. This boost represents the total system potential energy without the essential peptide potential energy included:

$$\Delta V_{D}\left(r\right)=\{\begin{array}{c} \frac{1}{2}k_{D}{\left(E_{D}-V_{D}\left(r\right)\right)}^{2},\;V_{D}\left(r\right)<E_{D}\\ 0,\;V_{D}\left(r\right)\geq E_{D} \end{array}$$ (7)

Where *V*_*D*_ represents the total system potential energy without the essential peptide potential energy included, *E*_*D*_ represents the second boost potential threshold energy and *k*_*D*_ represents the harmonic constant. Hence, this contributes to the dual-boost Pep-GaMD as the total boost potential $\Delta V\left(r\right)=\Delta V_{L}\left(r\right)+\Delta V_{D}\left(r\right)$ .

### Energetic Reweighting of Pep-GaMD Simulations

For energetic reweighting of Pep-GaMD simulations to calculate potential mean force (PMF), the probability distribution along a reaction coordinate is written as $p^{*}\left(A\right)$ . Given the boost potential ΔV(r) of each frame, $p^{*}\left(A\right)$ can be reweighted to recover the canonical ensemble distribution p(A) , as:

$$p\left(A_{j}\right)=p^{*}\left(A_{j}\right)\frac{{\langle e^{\beta \Delta V\left(r\right)}\rangle }_{j}}{\sum_{i=1}^{M}{\langle p^{*}\left(A_{i}\right)e^{\beta \Delta V\left(r\right)}\rangle }_{i}},\;j\;=\;1\text{,}...,M$$ (8)

where *M* is the number of bins, $\beta =k_{B}T$ and ${\langle e^{\beta \Delta V\left(r\right)}\rangle }_{j}$ is the ensemble-averaged Boltzmann factor of ΔV(r) for simulation frames found in the *j*th bin. The ensemble-averaged reweighting factor can be approximated using cumulant expansion:

$${\langle e^{\beta \Delta V\left(r\right)}\rangle }_{j}=\text{exp}\left\{\sum_{k=1}^{\infty }\frac{\beta^{k}}{k!}C_{k}\right\}$$ (9)

where first two cumulants are given by

$$C_{1}=\Delta V$$

$$C_{2}=\Delta V^{2}-\Delta V^{2}=\sigma_{V}^{2}$$ (10)

The boost potential obtained from Pep-GaMD simulations usually follows near-Gaussian distribution. Cumulant expansion to the second order thus provides a good approximation for computing the reweighting factor. The reweighted free energy $F\left(A\right)=-k_{B}T\text{ln}p\left(A\right)$ is calculated as

$$F\left(A\right)=F^{*}\left(A\right)-\sum_{k=1}^{2}\frac{\beta^{k}}{k!}C_{k}+F_{c}$$ (11)

where $F^{*}\left(A\right)=-k_{B}T\text{ln}p^{*}\left(A\right)$ is the modified free energy obtained from GaMD simulation and $F_{c}$ is a constant.

### Simulation Protocol

Computational models of CXCR4 bound to the peptide agonists, ASLW and RSVM, were built using the recently published computational model of the CXCR4 in complex with its endogenous ligand CXCL12 (Ngo et al., 2020). The two peptides were generated by mutating the first 4 residues of the CXCL12 to ASLW and RSVM, respectively, and then deleting the remaining residues 18–68 of CXCL12. Additionally, computational model of the CXCR4 receptor in complex with the wildtype 1–17 residues of CXCL12 (1–17wt) was also generated by removing residues 18–68 of CXCL12 (Supplementary Figure S1A). Control simulations were performed on the CXCL12 agonist-bound CXCR4 and the viral chemokine vMIP-II antagonist-bound CXCR4 (Qin et al., 2015). All simulation files have been made available at https://doi.org/10.6084/m9.figshare.18338603.

The CHARMM-GUI web server was used to prepare the peptide-bound CXCR4 receptor and embed the receptor in a POPC lipid bilayer (Supplementary Figure S1B). Neutral patches (acetyl and methylamide) were added to the protein termini residues. The peptide termini were kept as charged (NH3+ and COO-). The CHARMM36 m (Vanommeslaeghe and MacKerell, 2014) force field parameters were used for the protein, peptides and lipids. CHARMM-GUI output files and scripts were used with default parameters to prepare the systems for Pep-GaMD simulations. Energy minimization was performed for 5,000 steps using constant number, volume and temperature (NVT) ensemble at 310 K. Further equilibration was done for 375 ps at 310 K using NPT ensemble. Conventional MD (cMD) simulations was performed on the systems for 10 ns at 1 atm pressure and 310 K temperature. All-atom Pep-GaMD simulations were performed with a short cMD for 10 ns, Pep-GaMD equilibration for 55 ns followed by three independent Pep-GaMD production runs for 500 ns for each system. A cutoff distance of 9 Å was used for the van der Waals and short-range electrostatic interactions, and long-range electrostatic interactions were computed with the particle-mesh Ewald summation method (Darden et al., 1993). The simulation systems are ∼97 × 97 × 123 Å^3^ in dimension, containing a total of ∼90–100 K atoms with explicit solvent and lipid molecules.

Pep-GaMD trajectory analysis was performed using VMD (Humphrey et al., 1996) and CPPTRAJ (Roe and Cheatham, 2013) tools. All simulation trajectories for each system were combined for calculating reweighted free energy profiles using the PyReweighting (Miao et al., 2014) toolkit. Residue-residue interactions formed between the peptides and the CXCR4 receptor were investigated and the effect of residue interactions on the CXCR4 receptor activation regarding the distance between the intracellular ends of TM3 and TM6 of CXCR4 were examined over the simulation time course. A bin size of 1 Å was used for peptide-protein and TM3-TM6 distances, respectively. Free energy values were also reweighted for each of the peptide structural clusters. The cutoff was set to 500 frames in a bin or cluster for reweighting. Furthermore, structural clustering of the Pep-GaMD simulations was performed based on the peptide RMSD relative to the initial computational model to obtain top 10 representative peptide conformations in the receptor binding pocket using hierarchical agglomerative algorithm in CPPTRAJ.

## Results

Three independent 500 ns Pep-GaMD simulations were combined for each system and structural clustering was performed to obtain representative binding conformations of each peptide in the CXCR4. The top-ranked structural clusters of each system were analyzed and compared with known ligand-bound CXCR4 structures (Supplementary Figure S2A). The CXCR4 binding pocket is highly negatively charged allowing for strong interactions with positively charged amino acids of peptides or small-molecule atoms of the ligand (Roumen et al., 2012). Previous studies revealed the presence of two major and minor subpockets in the receptor orthosteric ligand-binding site (Roumen et al., 2012). While the small-molecule antagonist IT1t (PDB: 3ODU) and viral chemokine antagonist vMIP-II (PDB: 4RWS) bound to the minor subpocket involving interactions with residues D^2.63^ and E^7.39^ (Wu et al., 2010; Qin et al., 2015), the small cyclic peptide antagonist CVX15 (PDB: 3OE0) bound to the major subpocket involving residues D187^ECL2^ and D^6.58^ (Wu et al., 2010). Our previous study revealed that the plerixafor (PLX or AMD3100) drug bound both the major and minor subpockets of the orthosteric site (Pawnikar and Miao, 2020). In this context, the top-ranked binding conformations of the CXCL12 and 1–17wt peptides showed occupancy in both the major and minor subpockets (Supplementary Figures S2B, S2C) involving residues E^7.39^ from minor subpocket and D^6.58^ from major subpocket. In the top-ranked structural clusters, both ASLW and RSVM peptides interacted residues D^6.58^ and D^2.63^ from the major and minor subpockets, respectively (Supplementary Figures S2D, S2E). However, the RSVM peptide showed an additional interaction with residue E^7.39^ from the minor subpocket (Supplementary Figure S2E). Moreover, the positively charged N-terminus (NH_3_^+^) of each peptide could form salt-bridge interaction with residue Asp^2.63^ in the CXCR4 receptor (Supplementary Figure S3). Further analysis was performed regarding the important peptide-receptor interactions and the receptor intracellular TM3-TM6 distance for calculating the system free energy profiles.

### Viral Chemokine Antagonist vMIP-II Bound CXCR4 Sampled Inactive Low-Energy State

Three independent 500 ns Pep-GaMD simulations were performed on the high-resolution X-ray crystal structure of the CXCR4 bound to viral chemokine vMIP-II antagonist (Qin et al., 2015). Structural clustering of the Pep-GaMD simulation snapshots was performed using hierarchical agglomerative algorithm in CPPTRAJ. Top 10 representative peptide conformations based on the peptide RMSD relative to the initial computational model in the receptor binding pocket were obtained. In the top ranked structural cluster, residues R7, L1 (the backbone N atom) and G2 (the backbone N atom) in the vMIP-II peptide formed polar interactions with receptor residues D^6.58^, D^2.63^ and E^7.39^, respectively (Supplementary Figure S4D). Each of these residue distances was selected as one reaction coordinate and the distance between the Cα atoms of TM3 residue R^3.50^ and TM6 residue K^6.32^ as the second to calculate 2D free energy profiles. Only one low-energy state (“Inactive”) was identified from the free energy profiles as shown in Supplementary Figures S4A–C **,** in which the R^3.50^-K^6.32^ distance exhibited an energy minimum at ∼8 Å and ∼4 Å for the R7-D^6.58^, L1-D^2.63^, and G2-E^7.39^ peptide-protein residue interaction distances.

### CXCL12 Endogenous Agonist-Bound CXCR4 Sampled Active, Intermediate and Inactive Low-Energy States

Pep-GaMD simulations of the CXCR4-CXCL12 complex showed that peptide residues K1 and R8 formed polar interactions with CXCR4 residues E^7.39^ and D^6.58^, respectively (Supplementary Figures S5A–B). The distance between the charge centers of K1 (the NZ atom) and the E^7.39^ (the CD atom), and R8 (the CZ atom) and D^6.58^ (the CG atom) were used as one of the reaction coordinates to calculate 2D free energy profiles. The distance between the Cα atoms of TM3 residue R^3.50^ and TM6 residue K^6.32^ was used as the second reaction coordinate.

**FIGURE 1:**
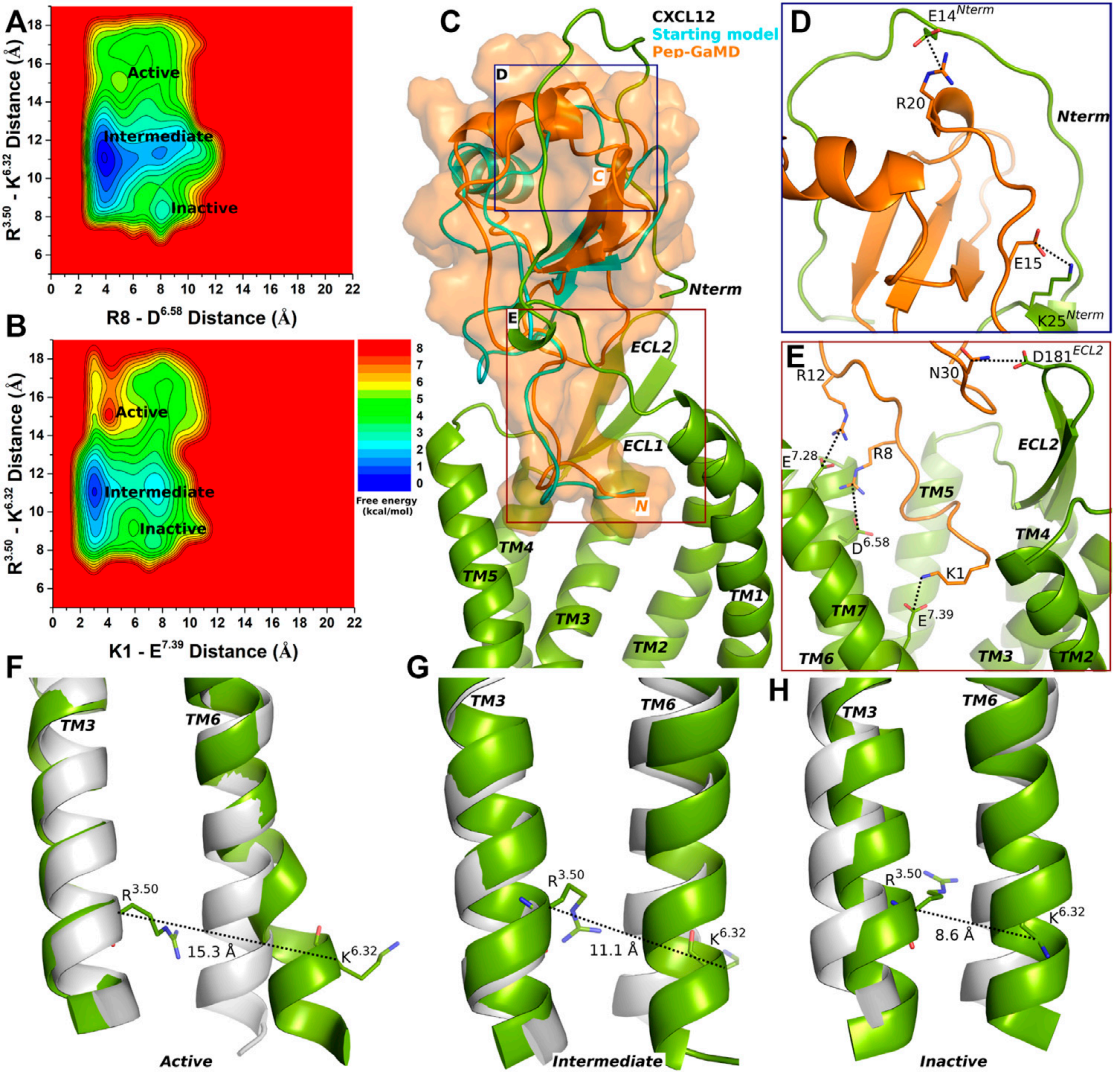
Endogenous agonist (CXCL12) bound CXCR4 sampled three Active, Intermediate and Inactive low-energy states. **(A–B)** Free energy profiles of CXCR4-CXCL12 interactions calculated regarding the distance between the Cα atoms of R^3.50^ and K^6.32^ and **(A)** distance between charge centers of peptide residue R8 (the CZ atom) and receptor residue D^6.58^ (the CG atom), **(B)** distance between charge centers of peptide residue K1 (the NZ atom) and receptor residue E^7.39^ (the CD atom). **(C)** Comparison of the top-ranked structural cluster of the endogenous agonist (CXCL12) obtained from Pep-GaMD simulations (orange cartoon) with the starting computational model (cyan cartoon). **(D–E)** Important residue interactions between the peptide (orange sticks) and receptor (green sticks) observed in the Pep-GaMD simulations. **(F–H)** The TM3-TM6 distance between the Cα atoms of R^3.50^ and K^6.32^ as observed in the **(F)** Active, **(G)** Intermediate and **(H)** Inactive states.

Three low-energy states Active, Intermediate and Inactive were identified from the free energy profiles as shown in Figure 1. The distance between the Cα atoms of residues R^3.50^ and K^6.32^ exhibited an energy minimum at ∼15.3 Å in the Active state, where salt bridges were also formed between residues R8-D^6.58^ and K1-E^7.39^ at ∼5 Å and ∼4 Å distance, respectively (Figures 1A,B,F). In the Intermediate state, a low-energy well was identified for the R^3.50^-K^6.32^ distance at ∼11.1 Å and the R8-D^6.58^ and K1-E^7.39^ salt-bridge distance at ∼4 Å and ∼3 Å, respectively (Figures 1A,B,G). In the Inactive state, the distance between the Cα atoms of R^3.50^-K^6.32^ exhibited an energy minimum at ∼8.6 Å and the R8-D^6.58^ and K1-E^7.39^ salt bridges were broken at a distance of ∼8 Å and 7.5 Å, respectively (Figures 1A,B,H).

The top-ranked binding conformation of CXCL12 obtained from Pep-GaMD simulations was compared with the starting computational model (Figure 1C). Important residue interactions were identified between CXCL12 and the N-terminus of the CXCR4 that looped around the peptide. Peptide residues E15 and R20 formed salt-bridge interactions with receptor N-terminus residues K25 and E14, respectively (Figure 1D). Along with the R8-D^6.58^ and K1-E^7.39^ residue interactions, additional contacts were identified for peptide residues R12 and N30 which formed ionic and polar interactions with CXCR4 residues E^7.28^ and D181^ECL2^, respectively (Figure 1E).

### 1–17 wt Peptide-bound CXCR4 Sampled Active and Intermediate Low-Energy States

**FIGURE 2:**
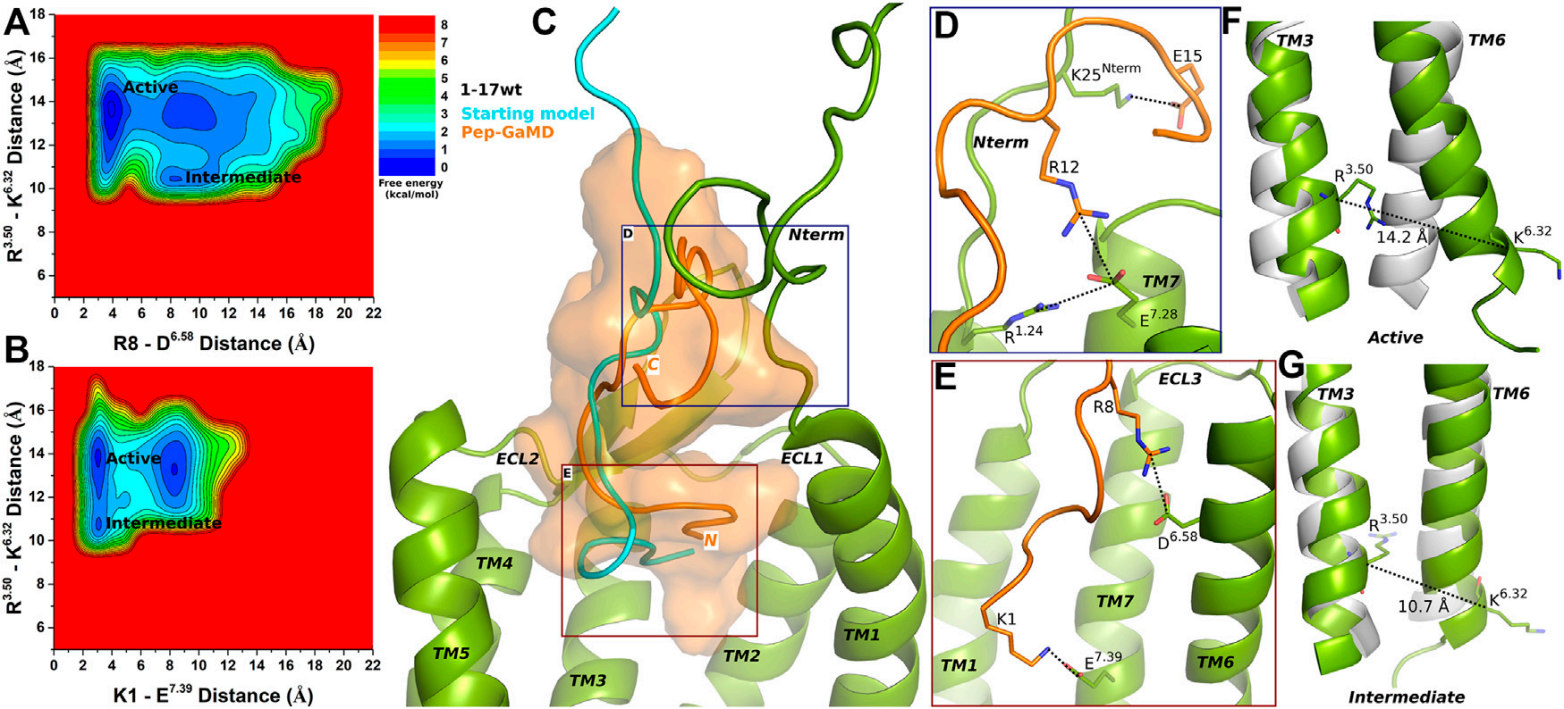
1–17 wt peptide (weak agonist) bound CXCR4 sampled Active and Intermediate low-energy states. **(A–B)** Free energy profiles of CXCR4-1–17wt interactions calculated regarding the distance between the Cα atoms of R^3.50^ and K^6.32^ and **(A)** distance between charge centers of peptide residue R8 (the CZ atom) and receptor residue D^6.58^ (the CG atom), **(B)** distance between charge centers of peptide residue K1 (the NZ atom) and receptor residue E^7.39^ (the CD atom). **(C)** Comparison of the top-ranked structural cluster of the 1–17wt peptide (weak agonist) obtained from Pep-GaMD simulations (orange cartoon) with the starting computational model (cyan cartoon). **(D–E)** Important residue interactions between the peptide (orange sticks) and receptor (green sticks) observed in the Pep-GaMD simulations. **(F–G)** The TM3-TM6 distance between the Cα atoms of R^3.50^ and K^6.32^ as observed in the **(F)** Active and the **(G)** Intermediate states.

Pep-GaMD simulations of the 1–17wt peptide-bound CXCR4 complex showed similar peptide-protein interactions as in the CXCR4-CXCL12 complex, i.e., between peptide residues K1 and R8 and CXCR4 residues E^7.39^ and D^6.58^, respectively (Supplementary Figures S6A–B). Two low-energy states Active and Intermediate were identified from the calculated 2D Free energy profiles (Figures 2A,B).

In the Active state, a low-energy well was identified at R^3.50^-K^6.32^ distance of ∼14.2 Å with the R8-D^6.58^ and K1-E^7.39^ salt-bridge forming at a distance of ∼3.5 Å and ∼3.3 Å, respectively (Figures 2A,B,F). The distance between the Cα atoms of residues R^3.50^ and K^6.32^ exhibited a low-energy minimum at ∼10.7 Å in the Intermediate state, where the R8-D^6.58^ salt-bridge was broken at a distance of ∼8 Å and the K1-E^7.39^ salt-bridge was formed at ∼3.3 Å, respectively (Figures 2A,B,G).

The binding conformation of 1–17 wt was significantly refined through Pep-GaMD simulations compared with the starting computational model (Figure 2C). The N-terminal residue K25 of CXCR4 formed a salt bridge with peptide residue E15 (Figure 2D). Additionally, receptor residue E^7.28^ was observed forming polar interactions with another receptor residue R^1.24^ and peptide residue R12, simultaneously (Figure 2D). Finally, the R8-D^6.58^ and K1-E^7.39^ residues formed salt-bridge interactions as described above (Figure 2E).

### Super Agonist ASLW-Bound CXCR4 Sampled Active and Intermediate Low-Energy Conformational States

**FIGURE 3:**
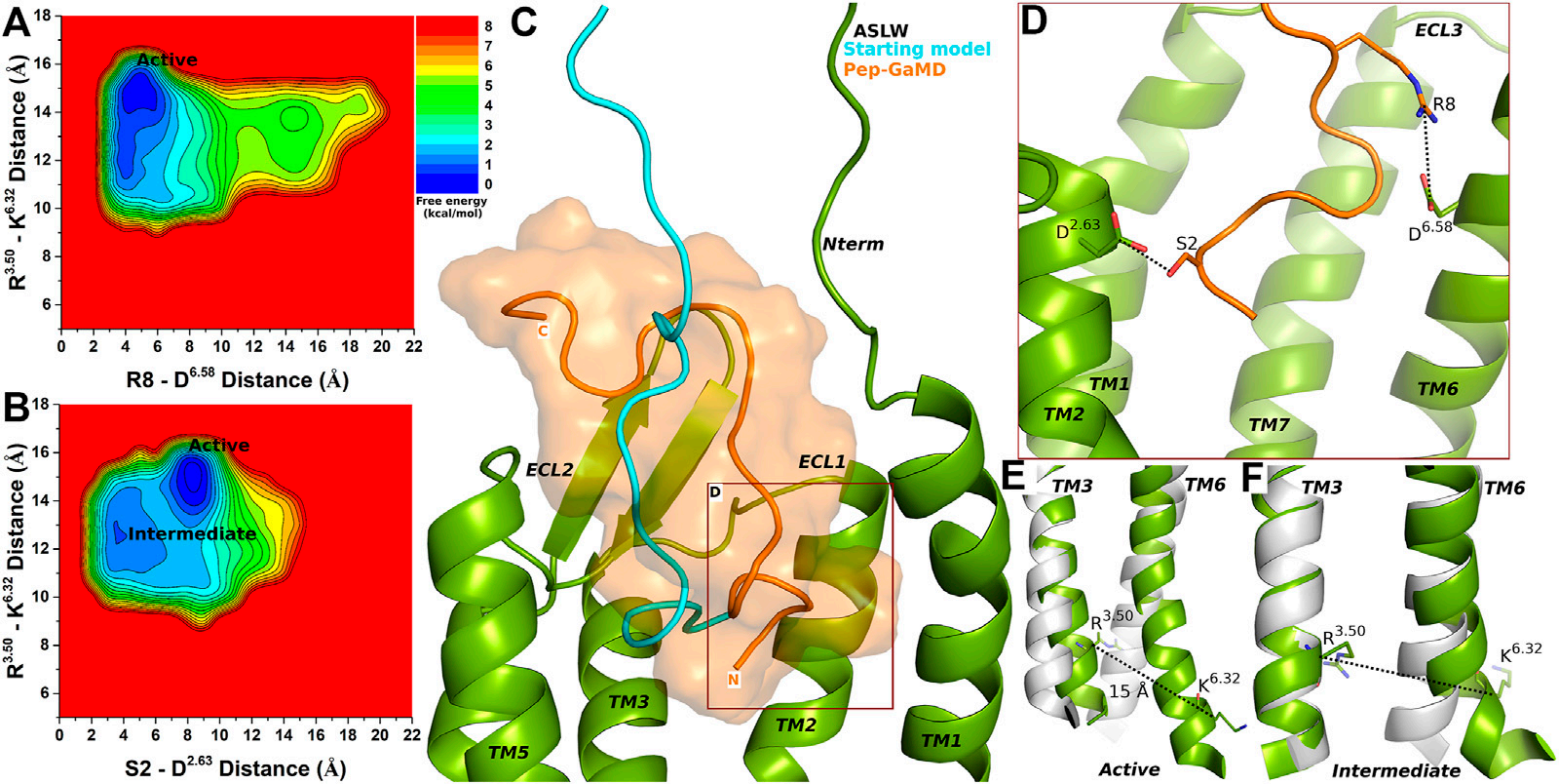
Super agonist ASLW bound CXCR4 sampled Active and Intermediate low-energy conformational states. **(A–B)** Free energy profiles of CXCR4-ASLW interactions calculated regarding the distance between the Cα atoms of R^3.50^ and K^6.32^ and **(A)** distance between charge centers of peptide residue R8 (the CZ atom) and receptor residue D^6.58^ (the CG atom), **(B)** distance between charge centers of peptide residue S2 (the OG atom) and receptor residue D^2.63^ (the CG atom). **(C)** Comparison of the top-ranked structural cluster of the super agonist ASLW obtained from Pep-GaMD simulations (orange cartoon) with the starting computational model (cyan cartoon). **(D)** Important residue interactions between the peptide (orange sticks) and receptor (green sticks) observed in the Pep-GaMD simulations. **(E–F)** The TM3-TM6 distance between the Cα atoms of R^3.50^ and K^6.32^ as observed in the **(E)** Active and the **(F)** Intermediate states.

In the top-ranked binding conformation, ASLW peptide residues S2 and R8 formed polar interactions with receptor residues D^2.63^ and D^6.58^, respectively (Supplementary Figures S7A–B and Figures 3A–D). Further analysis was done by calculating free energy profiles using the distance between the Cα atoms of R^3.50^ and K^6.32^ as one reaction coordinate and the peptide-protein polar interactions S2 (the OG atom)-D^2.63^ (the CG atom) or R8 (the CZ atom)-D^6.58^ (the CG atom) as the second reaction coordinate.

Two low-energy states Active and Intermediate were identified from the free energy profiles of ASLW bound-CXCR4 (Figures 3A,B), being similar to those in the 1–17wt bound-CXCR4 system. A low-energy well was identified for the Active state at ∼15 Å distance between the Cα atoms of residues R^3.50^ and K^6.32^ (Figures 3A,B,E). In the Active state, the R8-D^6.58^ salt-bridge could be formed at ∼4.8 Å distance (Figure 3A), however, the S2-D^2.63^ hydrogen bond was broken (Figure 3B). In the Intermediate state, the R^3.50^-K^6.32^ distance centered at ∼13 Å and residues S2-D^2.63^ formed a hydrogen bond at ∼3.5 Å distance (Figures 3B,F).

### Partial Agonist RSVM-Bound CXCR4 Sampled Intermediate and Inactive Low-Energy States

**FIGURE 4:**
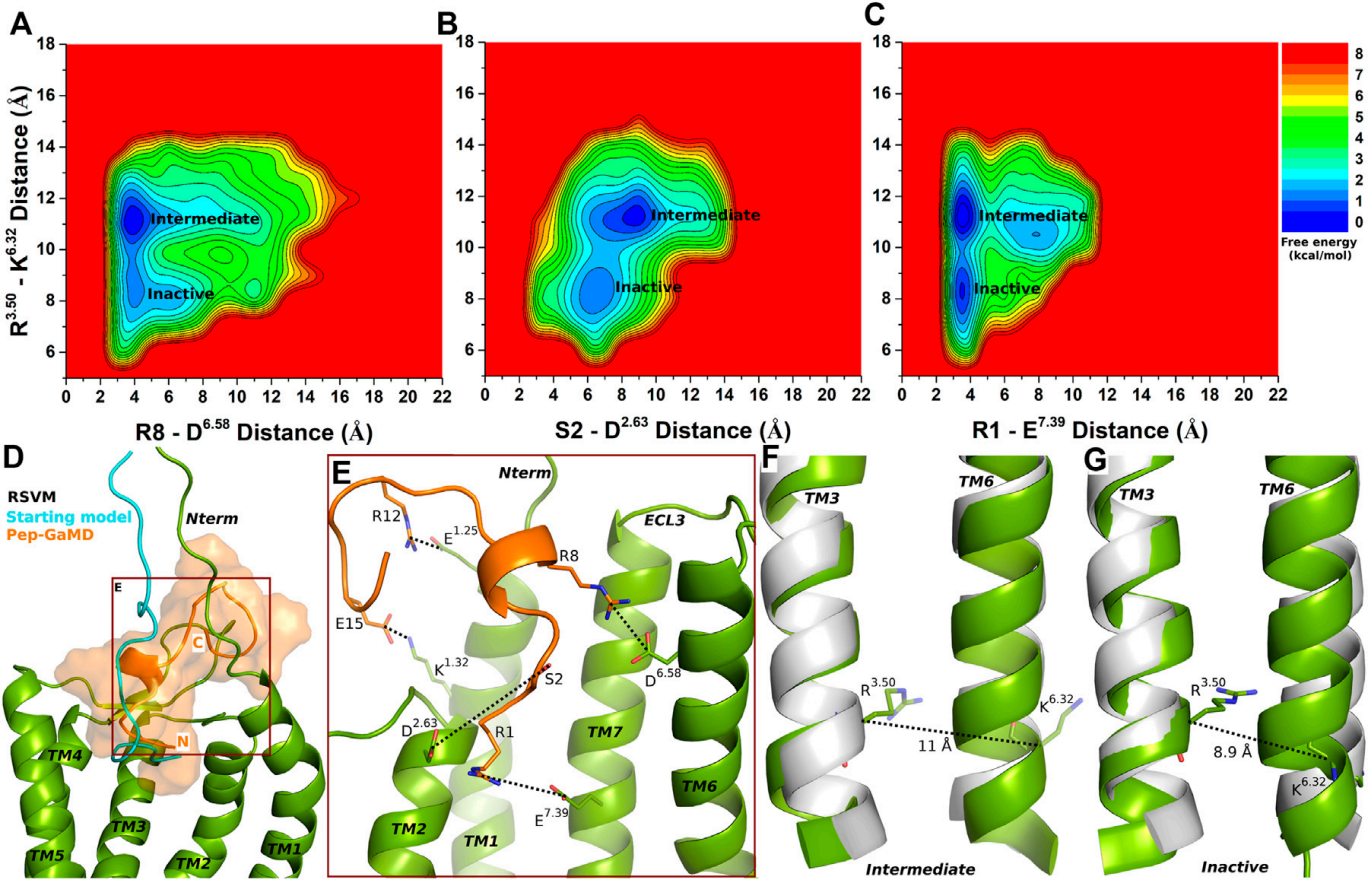
Partial agonist RSVM bound CXCR4 sampled Intermediate and Inactive low-energy states. **(A–C)** Free energy profiles of CXCR4-RSVM interactions calculated regarding the distance between the Cα atoms of R^3.50^ and K^6.32^ and **(A)** distance between charge centers of peptide residue R8 (the CZ atom) and receptor residue D^6.58^ (the CG atom), **(B)** distance between charge centers of peptide residue S2 (the OG atom) and receptor residue D^2.63^ (the CG atom), **(C)** distance between charge centers of peptide residue R1 (the CZ atom) and receptor residue E^7.39^ (the CD atom). **(D)** Comparison of the top-ranked structural cluster of the partial agonist RSVM obtained from Pep-GaMD simulations (orange cartoon) with the starting computational model (cyan cartoon). **(E)** Important residue interactions between the peptide (orange sticks) and receptor (green sticks) observed in the Pep-GaMD simulations. **(F–G)** The TM3-TM6 distance between the Cα atoms of R^3.50^ and K^6.32^ as observed in the **(F)** Intermediate and the **(G)** Inactive states.

Similar to the super agonist ASLW-bound CXCR4, the RSVM-CXCR4 complex exhibited polar interactions in the Pep-GaMD simulations between peptide residues S2 and R8 and receptor residues D^2.63^ and D^6.58^, respectively (Supplementary Figures S8A–B and Figures 4A–B). Additionally, another salt-bridge interaction was observed between the peptide residue R1 and CXCR4 residue E^7.39^ (Supplementary Figure S6C and Figure 4C). In the top-ranked structural cluster, residues R12 and E15 in the RSVM peptide also formed polar contacts with receptor residues E^1.25^ and K^1.32^ (Figures 4D,E). Residue distances between S2 (the OG atom) and D^2.63^ (the CG atom), R8 (the CZ atom) and D^6.58^ (the CG atom) and K1 (the NZ atom) and the E^7.39^ (the CD atom) were used as one reaction coordinate for calculating 2D free energy profiles. The distance between the Cα atoms of R^3.50^ and K^6.32^ was used as the second reaction coordinate.

The Intermediate and Inactive low-energy states were identified from free energy profiles of the RSVM bound CXCR4 as shown in Figure 4. In the Intermediate state, the R^3.50^-K^6.32^ distance centered at ∼11 Å (Figure 4F), the two salt-bridge interactions between R8-D^6.58^ and K1-E^7.39^ were formed at ∼4 Å distance (Figures 4A,C). The S2-D^2.63^ hydrogen bond, however, appeared broken in the Intermediate state at a distance of ∼8.3 Å (Figure 4B). In the Inactive state, the R^3.50^-K^6.32^ distance decreased to ∼8.9 Å (Figure 4G), the R8-D^6.58^ and K1-E^7.39^ formed polar interactions at ∼4 Å distance (Figures 4A,C). However, the S2-D^2.63^ hydrogen bond was broken at a distance of ∼6.5 Å (Figures 4B,E).

## Discussion

In this study, we have adopted a recently published computational model of the CXCR4-CXCL12 complex that was validated with cross-linking experimental data (Ngo et al., 2020). All-atom simulations using the novel Pep-GaMD method have been performed on the CXCR4 receptor bound to the ASLW, RSVM, 1–17wt, CXCL12 and vMIP-II peptides to refine the peptide-receptor complexes and capture the dynamic interactions between the peptides and CXCR4. Free energy profiles have been calculated for selected reaction coordinates through reweighting of the Pep-GaMD simulations.

It is important to note that the calculated free energy profiles are still not converged. For example, the endogenous agonist CXCL12 bound CXCR4 was able to sample the Active conformational state, although the probability appeared to be lower than that sampled by the 1–17 wt peptide agonist. This likely resulted from still insufficient sampling of the large conformational space of these highly flexible peptides despite enhanced sampling simulations using the Pep-GaMD method. In this regard, we calculated free energy profiles of individual Pep-GaMD simulations for each of the CXCL12, 1–17 wt, ASLW and RSVM bound CXCR4 systems, respectively (Supplementary Figures S10–S13). For the endogenous agonist CXCL12 bound CXCR4 system, while Sim1 sampled the “Inactive” state (Supplementary Figures S10A,D), both Sim1 and Sim2 sampled the “Active” state (Supplementary Figures S10A–B,D–E) and all three simulations sampled the “Intermediate” low-energy state (Supplementary Figures S10A–F). For the 1–17 wt weak agonist bound CXCR4, all three simulations sampled the “Active” low-energy state (Supplementary Figures S11A–F) and the “Intermediate” state was observed in both Sim2 and Sim3 (Supplementary Figures S11B–C,E–F). For the ASLW super agonist bound CXCR4, both Sim1 and Sim3 sampled the “Active” state (Supplementary Figures S12A,C,D,F) and all three simulations sampled the “Intermediate” state (Supplementary Figures S12A–F). Finally, for the RSVM partial agonist bound CXCR4, all the simulations sampled the “Intermediate” state (Supplementary Figures S13A–I) and Sim1 sampled the “Inactive” state (Supplementary Figures S13A,D,G **)**. Despite these variations, the free energy profiles of individual Pep-GaMD simulations showed similar results as compared with those calculated with all the simulations combined for each system. The discrete interactions between peptide agonists and CXCR4 highlighted the distinguished behavior of the peptide agonists. Important peptide-protein residue interactions and low-energy conformational states have been identified for each system from the Pep-GaMD simulations.

The orthosteric ligand-binding site of the CXCR4 receptor is divided into the major and minor subpockets involving negatively charged residues namely D^2.63^ (minor subpocket), D^6.58^ and E^7.39^ (major subpocket). These residues formed important interactions with positively charged atoms/residues in known antagonists such as small molecule (IT1t) (Wu et al., 2010), viral chemokine (vMIP-II) (Qin et al., 2015) and small cyclic peptide (CVX15) (Wu et al., 2010). Our previous study on the PLX drug binding to CXCR4 receptor revealed a novel intermediate binding site, which involved polar residues in the ECL2-TM5-TM6 region of the CXCR4 namely D187^ECL2^, D^5.32^ and D^6.58^ of CXCR4 (Pawnikar and Miao, 2020).

In previous studies, super agonist ASLW displayed a chemotactic index greater than the maximum identified in CXCL12 (or SDF-1α), while partial agonist RSVM displayed a down-regulation of surface CXCR4 (Sachpatzidis et al., 2003). Mutations of the first four residues in the full-length CXCL12 (or SDF-1α) to RSVM also generated a partial agonist of CXCR4, indicating that the peptide bound to the orthosteric site (Sachpatzidis et al., 2003). Our Pep-GaMD simulations revealed similar interactions of positively charged residues in the CXCL12, 1–17 wt and RSVM peptides with receptor residues E^7.39^ and D^6.58^ in the orthosteric site. In the Pep-GaMD simulations, interaction was also observed between peptide residue R8 and receptor residue D^6.58^ located in the major subpocket, which overlapped with the intermediate binding site of the PLX small-molecule drug (Pawnikar and Miao, 2020). This suggested a critical role of residue D^6.58^ in binding and activation of the CXCR4.

The differentiated agonism of our studied peptide ligands apparently resulted from the distinct interactions between the peptide N-terminal residues with the CXCR4 and different free energy landscapes of the CXCR4-peptide complexes. Importantly, residue E^7.39^ situated deeply in the binding pocket formed a hydrogen bond interaction with peptide residues G2 in the vMIP-II antagonist and salt-bridge with positively charged R1 in the RSVM partial agonist, and K1 in the 1–17 wt and CXCL12 agonists. On the contrary, such interaction was absent for the ASLW super agonist. Therefore, the absence of polar interaction with receptor residue E^7.39^ apparently contributed to the super agonism activity of the ASLW peptide, while the presence of such interaction could lead to reduced agonism activities of the CXCL12, 11–17 wt and RSVM peptides and the antagonism activity of the vMIP-II peptide. Overall, the CXCL12 endogenous agonist-bound CXCR4 sampled a large conformational space covering the active, intermediate and inactive states. While the super agonist biased the receptor towards the active and intermediate states, the partial agonist biased the receptor to the intermediate and inactive states and the antagonist stabilized the receptor mostly in the inactive state.

In conclusion, we determined the mechanism of peptide agonist binding in the CXCR4 receptor through accelerated molecular simulations using the novel Pep-GaMD technique. The mechanistic insights into structural dynamics and agonism of different peptides have provided a significant framework for design and development of new peptide modulators of the CXCR4 and other chemokine receptors.

## Data Availability

The datasets presented in this study can be found in online repositories. The names of the repository/repositories and accession number(s) can be found in the article/Supplementary Material.
