# Supplementary material for: Mechanism of Peptide Agonist Binding in CXCR4 Chemokine Receptor
Source: Front Mol Biosci. 2022 Mar 11;9:821055. doi: 10.3389/fmolb.2022.821055 (PMC8963245; doi:10.3389/fmolb.2022.821055)
Supplement: Supplementary file 1 [file DataSheet1.PDF]

Supporting Information

Mechanism of Peptide Agonist Binding in CXCR4 Chemokine Receptor

Shristi Pawnikar and Yinglong Miao*

Center for Computational Biology and Department of Molecular Biosciences, University of Kansas, Lawrence, KS 66047, USA

Email: miao@ku.edu

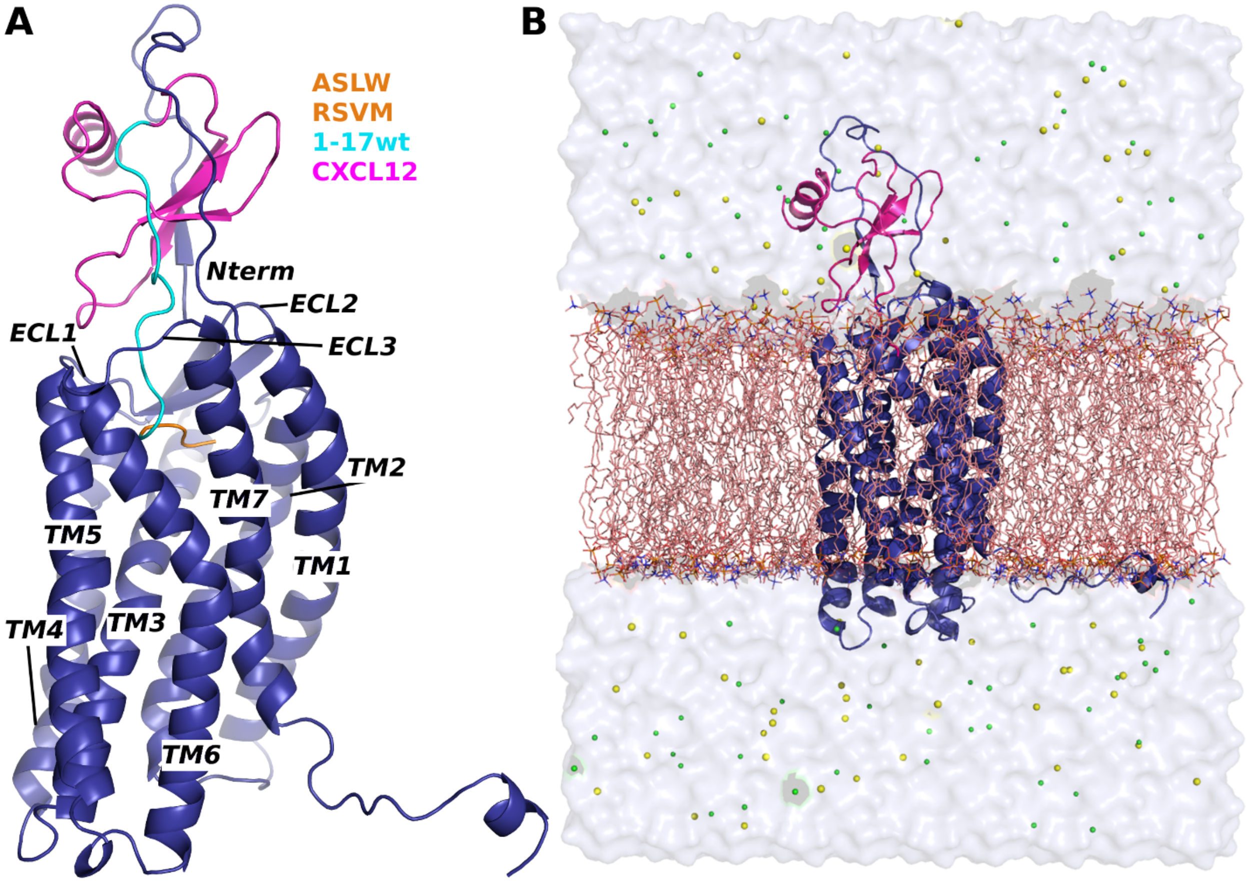

Figure S1. (**A**) Computational model of CXCR4 (blue cartoon) in complex with endogenous ligand CXCL12 (magenta cartoon) used to generate 1-17wt (cyan) and the mutant peptides ASLW and RSVM (orange) bound CXCR4 systems. (**B**) Example Pep-GaMD simulation system of the CXCR4-CXCL12 complex with the receptor embedded in a POPC lipid bilayer (orange sticks) and complex solvated in 0.15 M NaCl solution.

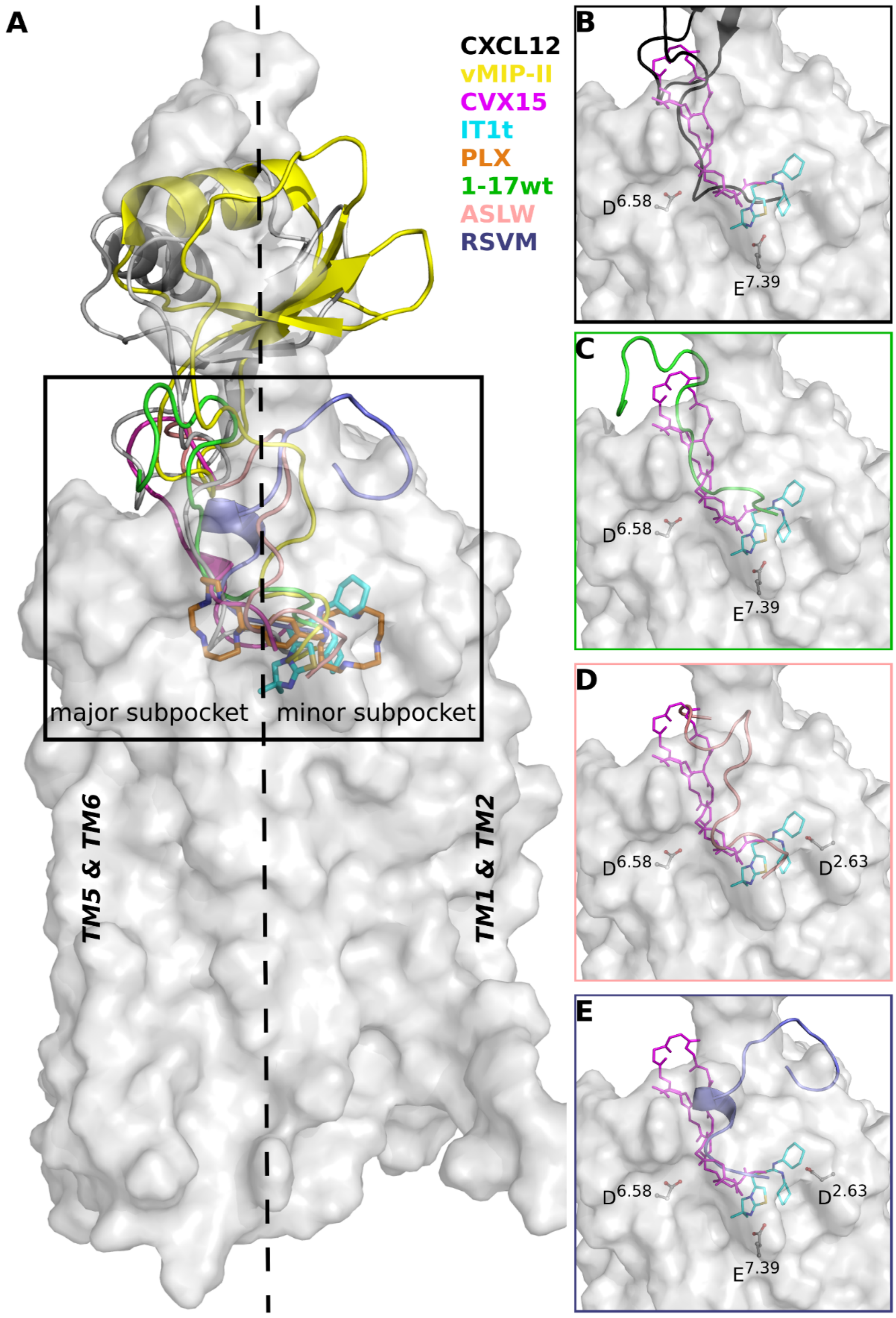

Figure S2. Comparison of peptide binding conformations obtained through Pep-GaMD simulations with X-ray conformations of known antagonists in CXCR4. (**A**) Global view of

Pep-GaMD simulation predicted binding conformations of CXCL12 (black), 1-17wt (green), ASLW (pink) and RSVM (blue) peptides in the CXCR4 receptor (white surface) compared with X-ray crystal conformations of viral chemokine vMIP-II (yellow), cyclic peptide CVX15 (magenta) and small molecules IT1t (cyan) and PLX (orange). The ligand binding pocket is divided into major and minor subpockets along the axis shown in dashed lines. Enlarged view of the binding conformations of (**B**) CXCL12, (**C**) 1-17wt, (**D**) ASLW, and (**E**) RSVM as compared with antagonists occupying the major subpocket (CVX15) and minor subpocket (IT1t).

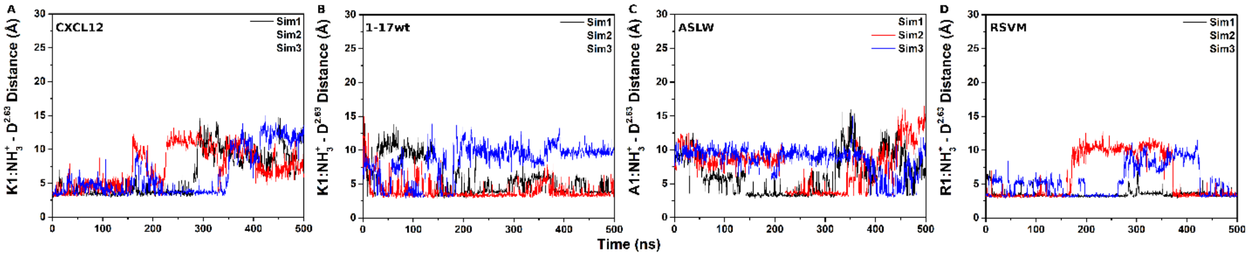

Figure S3. (**A-D**) Time courses of the interaction between the charged N-terminus of the peptides and CXCR4 residue $D^{2.63}$ observed in Pep-GaMD simulations.

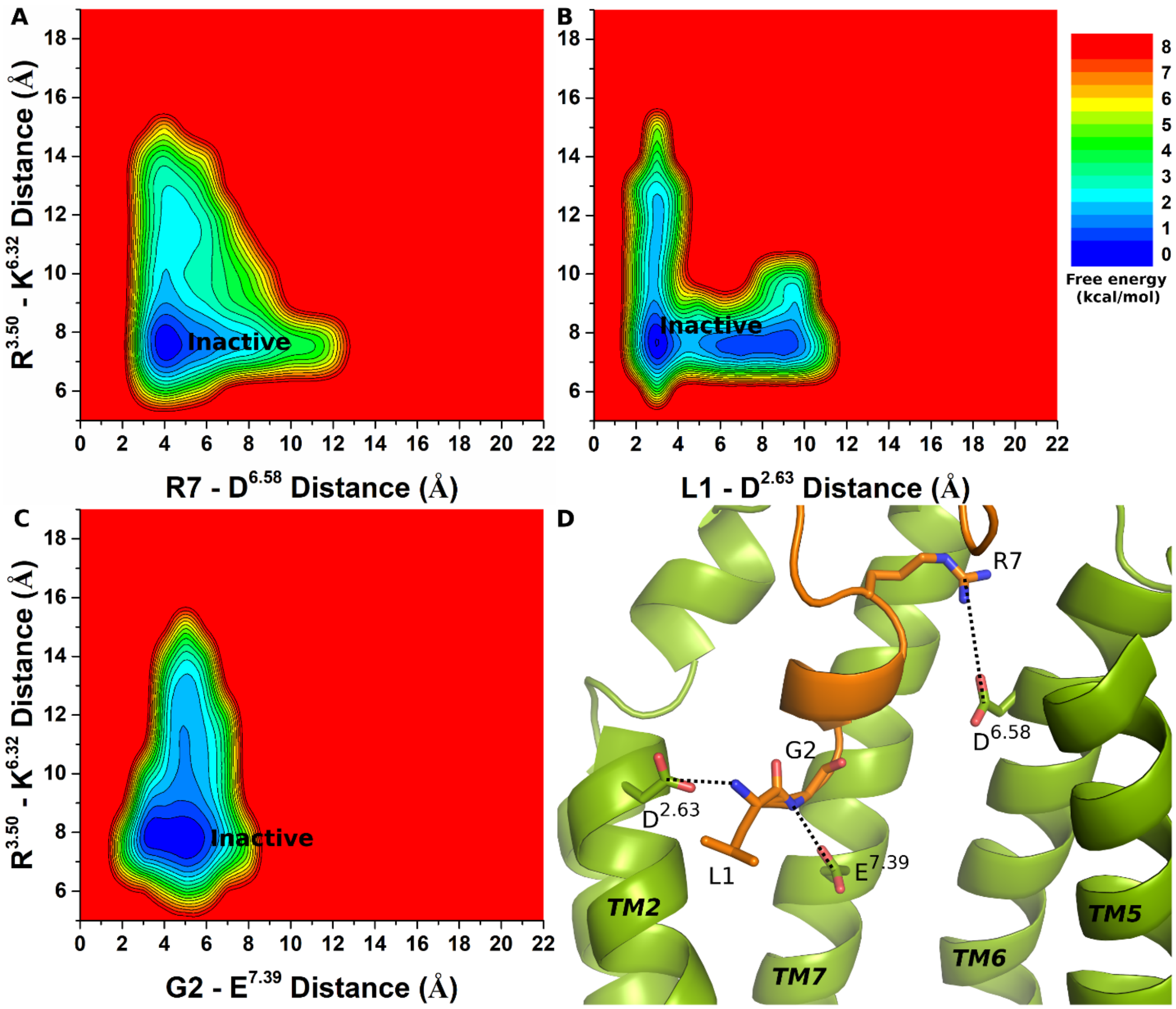

Figure S4. (**A-C**) Free energy profiles of CXCR4-vMIP-II interactions calculated regarding the distance between the Cα atoms of $R^{3.50}$ and $K^{6.32}$ and (**A**) distance between charge centers of peptide residue R7 (the CZ atom) and receptor residue $D^{6.58}$ (the CG atom), (**B**) distance between charge centers of peptide residue L1 (the N atom) and receptor residue $D^{2.63}$ (the CG atom), (**C**) distance between charge centers of peptide residue G2 (the N atom) and receptor residue $E^{7.39}$ (the CD atom). (**D**) Important residue interactions between the peptide (orange sticks) and receptor (green sticks) observed in the Pep-GaMD simulations.

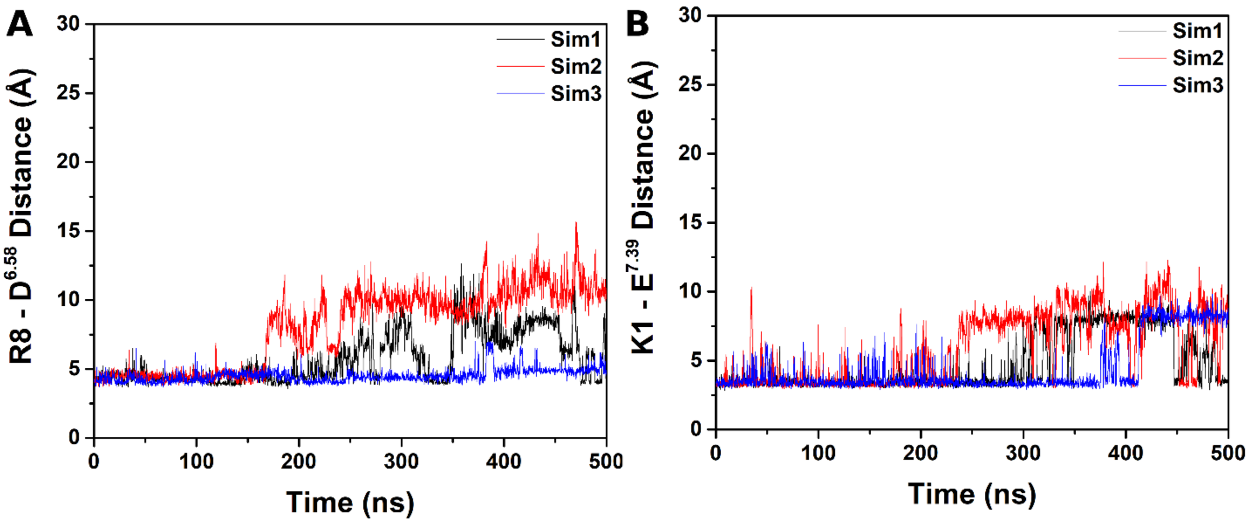

Figure S5. Time courses of interactions observed in the CXCL12 bound CXCR4 Pep-GaMD simulations between (**A**) peptide residue R8 (the CZ atom) and CXCR4 residue $D^{6.58}$ (the CG atom), and (**B**) peptide residue K1 (the NZ atom) and CXCR4 residue $E^{7.39}$ (the CG atom).

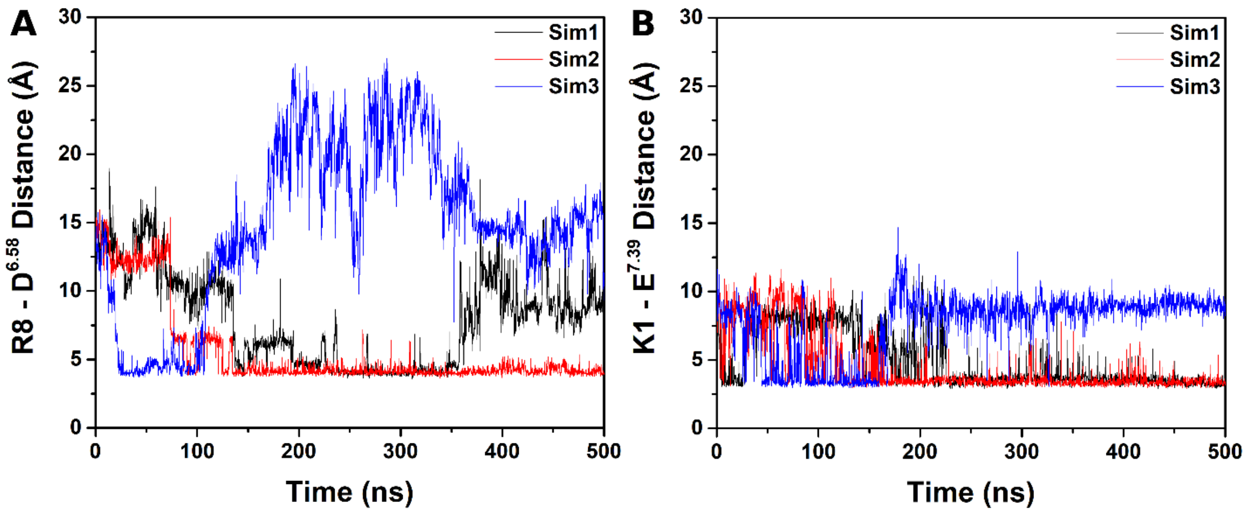

Figure S6. Time courses of interactions observed in the 1-17wt bound CXCR4 Pep-GaMD simulations between (**A**) peptide residue R8 (the CZ atom) and CXCR4 residue $D^{6.58}$ (the CG atom), and (**B**) peptide residue K1 (the NZ atom) and CXCR4 residue $E^{7.39}$ (the CG atom).

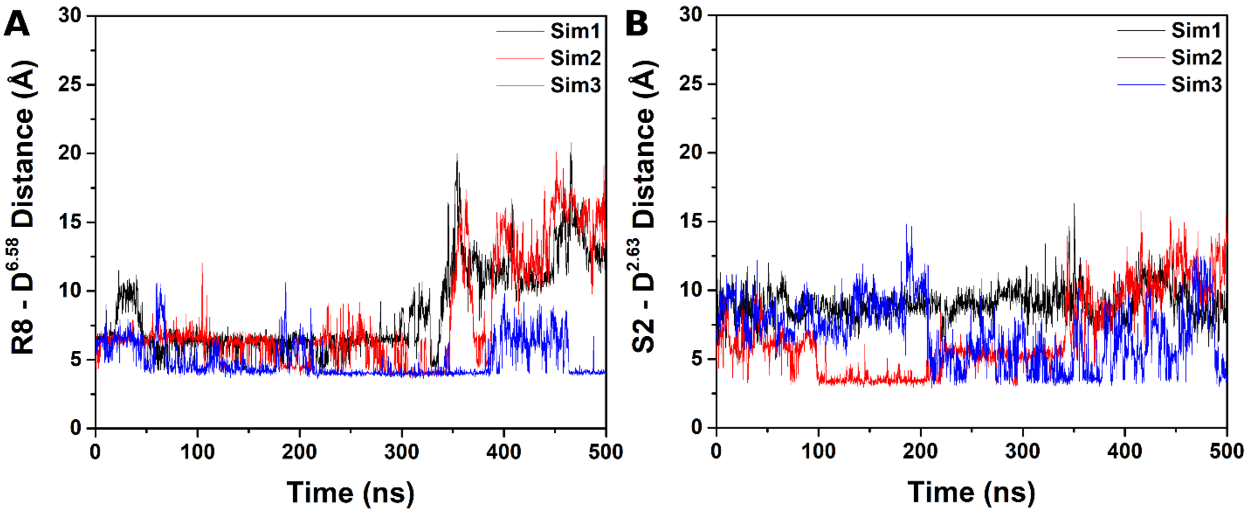

Figure S7. Time courses of interactions observed in the ASLW bound CXCR4 Pep-GaMD simulations between (**A**) peptide residue R8 (the CZ atom) and CXCR4 residue $D^{6.58}$ (the CG atom), and (**B**) peptide residue S2 (the OG atom) and CXCR4 residue $D^{2.63}$ (the CG atom).

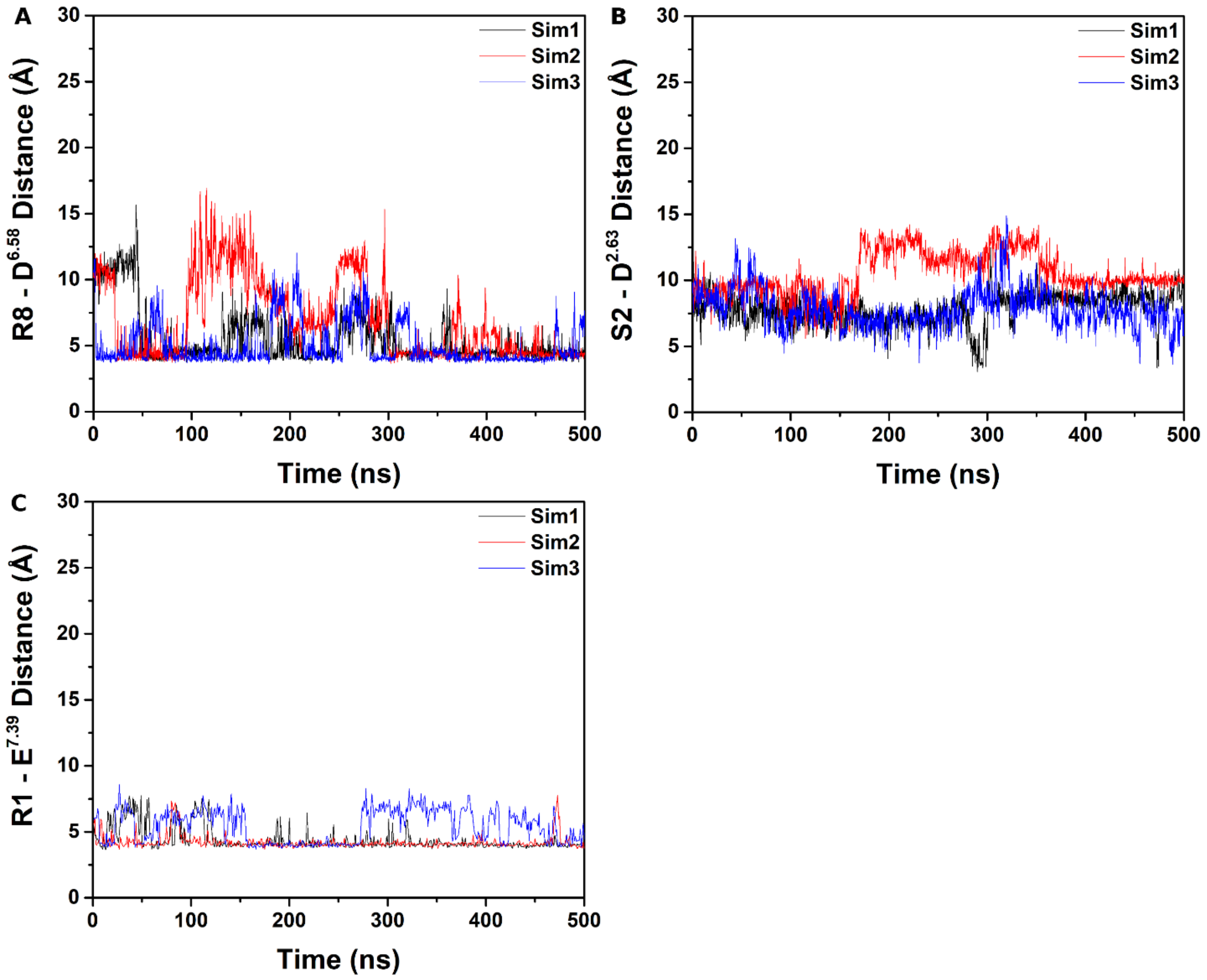

Figure S8. Time courses of interactions observed in the RSVM bound CXCL12 Pep-GaMD simulations between (**A**) peptide residue R8 (the CZ atom) and CXCR4 residue $D^{6.58}$ (the CG atom), (**B**) peptide residue S2 (the OG atom) and CXCR4 residue $D^{2.63}$ (the CG atom), and (**C**) peptide residue R1 (the CZ atom) and CXCR4 residue $E^{7.39}$ (the CG atom).

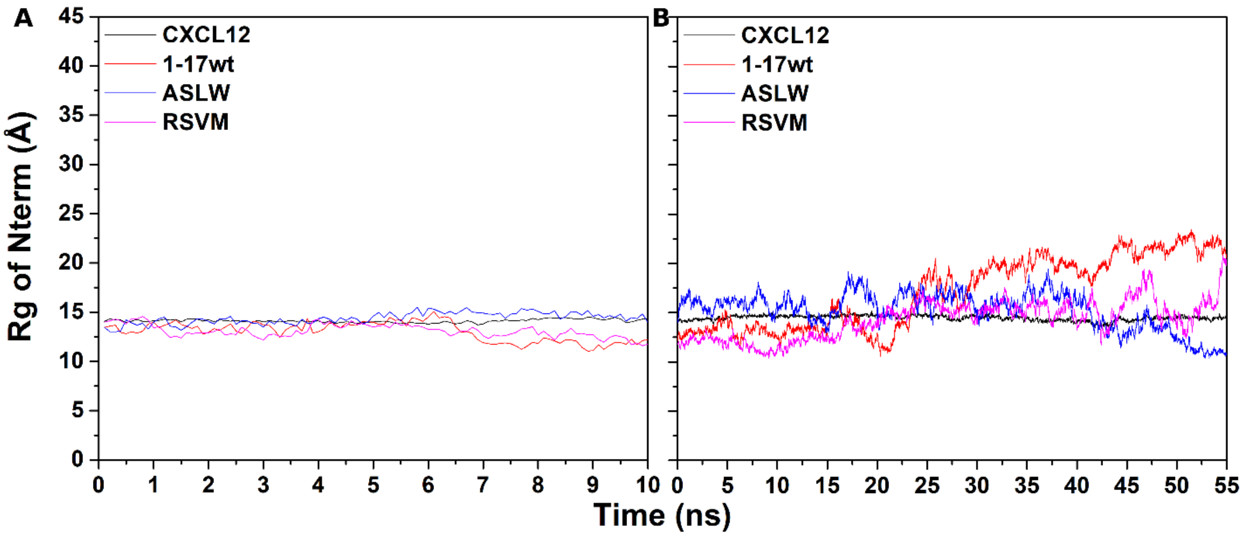

Figure S9. Time course plots of radius of gyration (Rg) of the CXCR4 N-terminus observed in (**A**) cMD and (**B**) Pep-GaMD equilibration.

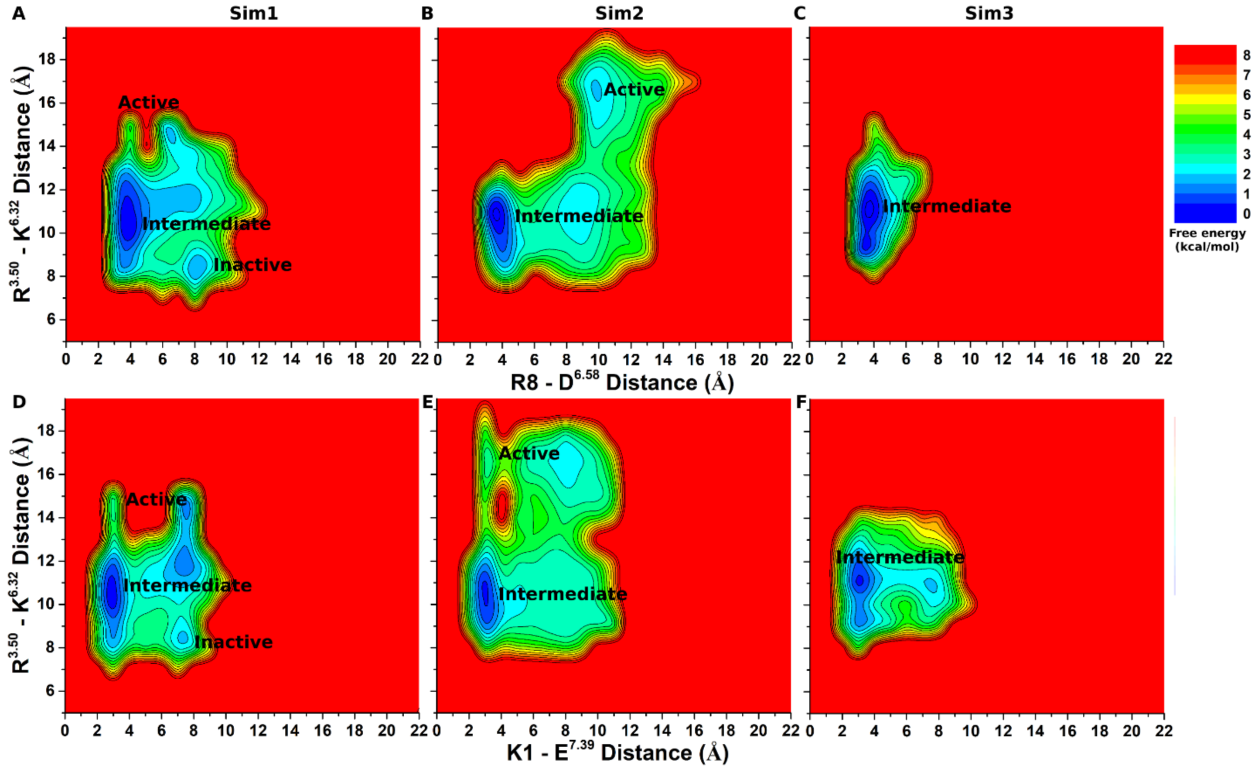

Figure S10. Free energy profiles of CXCR4-CXCL12 interactions regarding the distance between the Cα atoms of $R^{3.50}$ and $K^{6.32}$ and (**A-C**) distance between charge centers of peptide residue R8 (the CZ atom) and receptor residue $D^{6.58}$ (the CG atom), and (**D-F**) distance between charge centers of peptide residue K1 (the NZ atom) and receptor residue $E^{7.39}$ (the CD atom) calculated from (**A,D**) Sim1, (**B,E**) Sim2, and (**C,F**) Sim3 of the Pep-GaMD simulations. Important low-energy conformational states are identified, including the “Active”, “Intermediate” and “Inactive”.

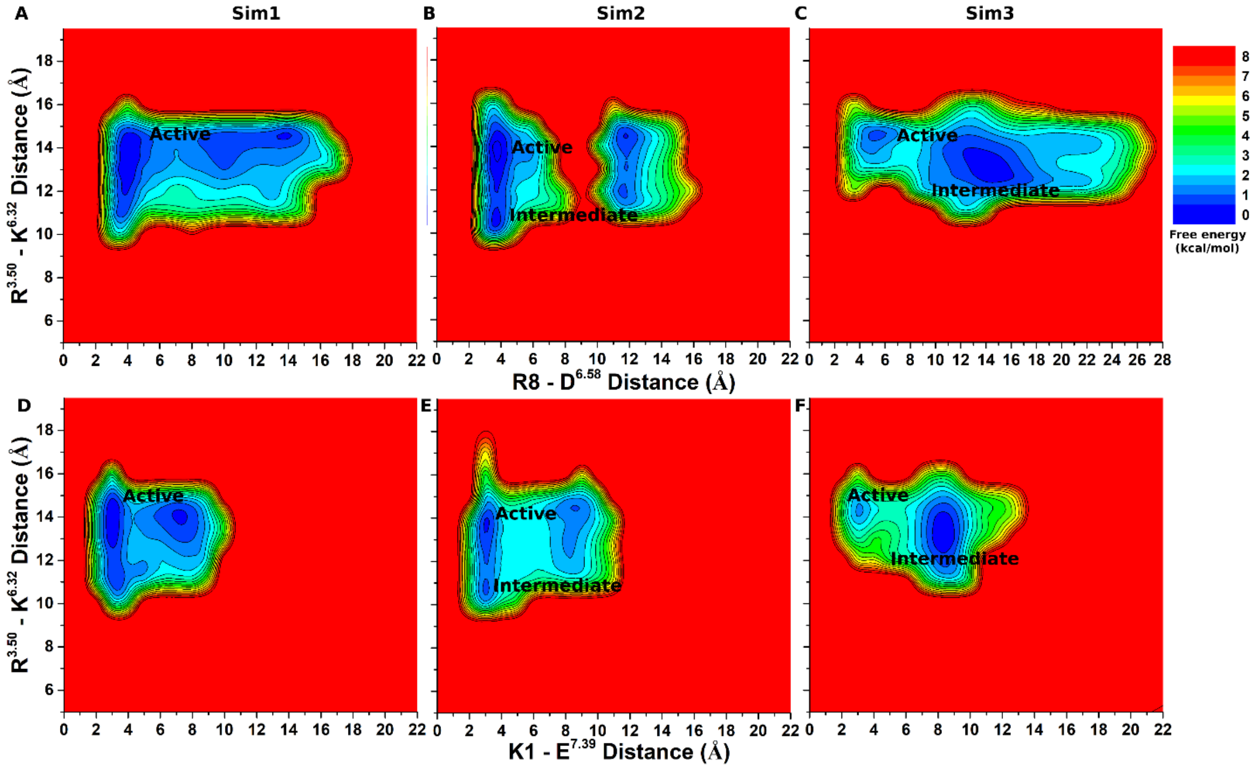

Figure S11. Free energy profiles of CXCR4-1-17wt interactions regarding the distance between the Cα atoms of $R^{3.50}$ and $K^{6.32}$ and (**A-C**) distance between charge centers of peptide residue R8 (the CZ atom) and receptor residue $D^{6.58}$ (the CG atom), and (**D-F**) distance between charge centers of peptide residue K1 (the NZ atom) and receptor residue $E^{7.39}$ (the CD atom) calculated from (**A,D**) Sim1, (**B,E**) Sim2, and (**C,F**) Sim3 of the Pep-GaMD simulations. Important low-energy conformational states are identified, including the "Active" and "Intermediate".

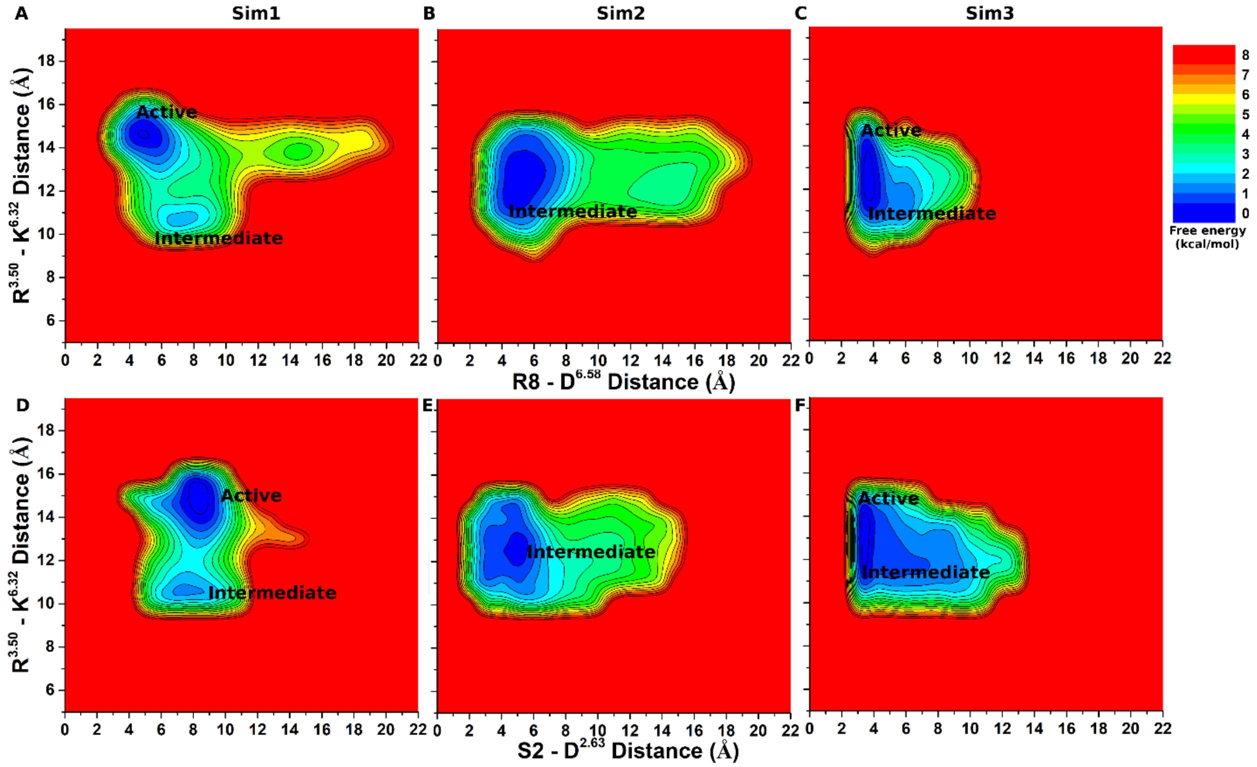

Figure S12. Free energy profiles of CXCR4-ASLW interactions regarding the distance between the Cα atoms of $R^{3.50}$ and $K^{6.32}$ and (**A-C**) distance between charge centers of peptide residue R8 (the CZ atom) and receptor residue $D^{6.58}$ (the CG atom), and (**D-F**) distance between charge centers of peptide residue S2 (the OG atom) and receptor residue $D^{2.63}$ (the CG atom) calculated from (**A,D**) Sim1, (**B,E**) Sim2, and (**C,F**) Sim3 of the Pep-GaMD simulations. Important low-energy conformational states are identified, including the "Active" and "Intermediate".

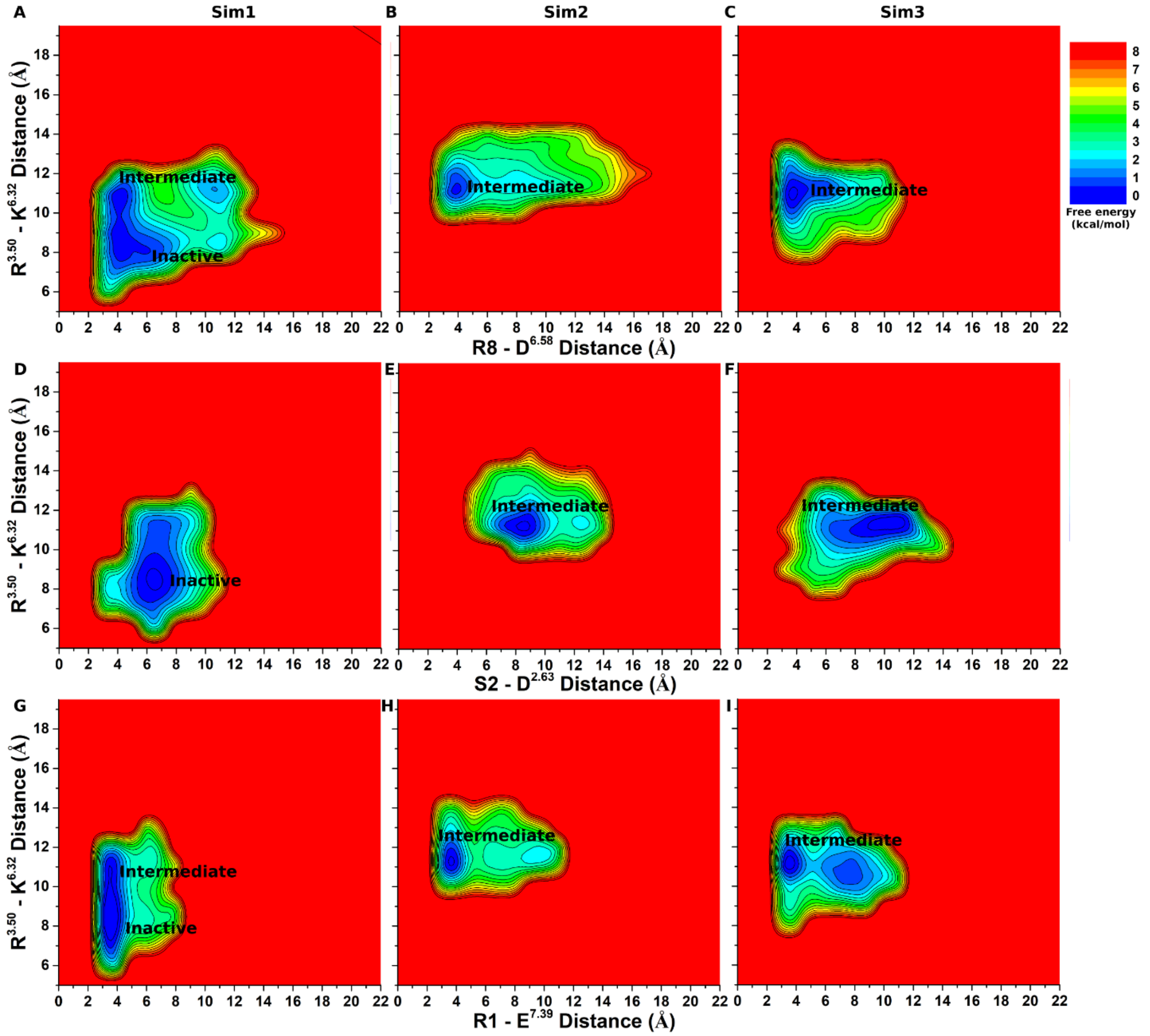

Figure S13. Free energy profiles of CXCR4-RSVM interactions regarding the distance between the Cα atoms of $R^{3.50}$ and $K^{6.32}$ and (**A-C**) distance between charge centers of peptide residue R8 (the CZ atom) and receptor residue $D^{6.58}$ (the CG atom), (**D-F**) distance between charge centers of peptide residue S2 (the OG atom) and receptor residue $D^{2.63}$ (the CG atom), and (G-I) distance between charge centers of peptide residue R1 (the CZ atom) and receptor residue $E^{7.39}$ (the CD atom) calculated from (**A,D,G**) Sim1, (**B,E,H**) Sim2, and (**C,F,I**) Sim3 of the Pep-GaMD simulations. Important low-energy conformational states are identified, including the "Inactive" and "Intermediate".
